# Real-world effectiveness of post-trastuzumab emtansine treatment in patients with HER2-positive, unresectable and/or metastatic breast cancer: a retrospective observational study (KBCSG-TR 1917)

**DOI:** 10.1186/s12885-021-08504-1

**Published:** 2021-07-09

**Authors:** Takahiro Nakayama, Tetsuhiro Yoshinami, Hiroyuki Yasojima, Nobuyoshi Kittaka, Masato Takahashi, Shoichiro Ohtani, Seung Jin Kim, Hiroyuki Kurakami, Naoko Yamamoto, Tomomi Yamada, Takehiko Takata, Norikazu Masuda

**Affiliations:** 1 Department of Breast and Endocrine Surgery, Osaka International Cancer Institute, 3-1-69, Otemae, Chuo-ku, Osaka, 541-8567 Japan; 2 Department of Breast and Endocrine Surgery, Graduate School of Medicine, Osaka University, 2-2-E-10 Yamadaoka, Suita City, Osaka, 565-0871 Japan; 3 Department of Surgery, Breast Oncology, National Hospital Organization Osaka National Hospital, 2-1-14, Hoenzaka, Chuo-ku, Osaka, 540-0006 Japan; 4 Department of Breast Surgery, National Hospital Organization Hokkaido Cancer Center, 2-3-54, Kikusui 4-jo Shiroishi-ku, Sapporo, Hokkaido 003-0804 Japan; 5 Department of Breast Surgery, Hiroshima City Hiroshima Citizens Hospital, 7-33 Motomachi, Naka-ku, Hiroshima, 730-8518 Japan; 6 Present address: Ohtani Shoichiro Breast Clinic, 4-18-101, Hatchobori, Naka-ku, Hiroshima, 730-0013 Japan; 7 Department of Medical Innovation, Osaka University Hospital, 2-15, Yamadaoka, Suita, Osaka, 565-0871 Japan; 8 Oncology Medical Science Department, Daiichi Sankyo Co., Ltd., 3-5-1, Nihonbashi-honcho, Chuo-ku, Tokyo, 103-8426 Japan

**Keywords:** Retrospective observational study, HER2-positive, Unresectable and/or metastatic breast cancer, KBCSG-TR 1917, T-DM1/trastuzumab emtansine

## Abstract

**Background:**

Trastuzumab emtansine (T-DM1) is a second-line standard therapy for patients with human epidermal growth factor receptor 2 (HER2)-positive metastatic breast cancer. Evidence regarding post–T-DM1 treatments is currently lacking. We evaluated the effectiveness of post–T-DM1 drug therapy in patients with HER2-positive, unresectable and/or metastatic breast cancer.

**Methods:**

In this multicenter, retrospective, observational study, real-world clinical data of female patients with HER2-positive breast cancer who had a history of T-DM1 treatment were consecutively collected from five sites in Japan. We investigated the effectiveness of post–T-DM1 therapy by evaluating the real-world progression-free survival (rwPFS), time to treatment failure (TTF), overall survival (OS), objective response rate (ORR), and clinical benefit rate (CBR). Tumor response was assessed by investigators according to Response Evaluation Criteria in Solid Tumors (RECIST version 1.1) guidelines. Subgroup and exploratory analyses according to background factors were also undertaken.

**Results:**

Of the 205 patients who received T-DM1 treatment between 1 January 2014 and 31 December 2018, 128 were included in this study. Among the 128 patients analyzed, 105 (82%) patients received anti-HER2 therapy and 23 (18%) patients received regimens without anti-HER2 therapy. Median (95% confidence interval [CI]) rwPFS, TTF, and OS were 5.7 (4.8–6.9) months, 5.6 (4.6–6.4) months, and 22.8 (18.2–32.4) months, respectively. CBR and ORR (95% CI) were 48% (38.8–56.7) and 23% (15.1–31.4), respectively. Cox-regression analysis showed that an ECOG PS score of 0, a HER2 immunohistochemistry score of 3+, recurrent type, ≥12 month duration of T-DM1 therapy, and anti-HER2 therapy were independent variables for rwPFS. An exploratory subgroup analysis of regimens after T-DM1 showed that those with anti-HER2 therapy had a median rwPFS of 6.3 and those without anti-HER2 therapy had a median rwPFS of 4.8 months.

**Conclusions:**

In the real-world setting in Japan, several post–T-DM1 regimens for patients with unresectable and/or metastatic HER2-positive breast cancer, including continuation of anti-HER2 therapy, showed some effectiveness; however, this effectiveness was insufficient. Novel therapeutic options are still needed for further improvement of PFS and OS in later treatment settings.

**Trial registration:**

UMIN000038296; registered on 15 October 2019.

**Supplementary Information:**

The online version contains supplementary material available at 10.1186/s12885-021-08504-1.

## Background

Human epidermal growth factor receptor 2 (*HER2*) is a growth factor receptor gene that is amplified in approximately 15–20% of breast cancers, and HER2 protein overexpression on the plasma membrane of tumor cells reportedly correlates with a poor prognosis [1–6].

Trastuzumab, a HER2-targeting monoclonal antibody, was approved in 1998 and has improved outcomes in patients with HER2-positive breast cancer [7, 8]. Following trastuzumab approval, other HER2-targeted drugs have subsequently been approved for use in these patients, including lapatinib [9, 10] and pertuzumab [11, 12]. These therapies have been reported to prolong progression-free survival (PFS) and overall survival (OS), and to be more efficacious than conventional chemotherapies [11–16]. Based on the outcomes of the CLEOPATRA trial [12], pertuzumab + trastuzumab + taxane is currently recommended as first-line therapy in HER2-positive metastatic breast cancer [17].

The EMILIA trial investigated the use of trastuzumab emtansine (T-DM1) as a second-line therapeutic to follow treatment with trastuzumab and a taxane [13]. Additionally, the TH3RESA trial demonstrated that patients who had previously been treated with two or more regimens experienced an increase in objective response rate (ORR) and a prolongation of PFS and OS with T-DM1 treatment [14, 15]. T-DM1 is now the standard of care for patients with HER2-positive metastatic breast cancer who were previously treated with trastuzumab + taxane [18]. However, the development of T-DM1 resistance, either through reduced HER2 expression, reduced T-DM1 binding, or other subversive signaling abnormalities, remains a challenge [19]. For example, we previously reported several cases where tumors became HER2-negative after T-DM1 treatment [20]. Currently, there are no established treatment options to follow T-DM1 therapy that have shown adequate evidence in real-world settings. Therefore, we planned the present study to establish real-world evidence to support clinical treatment decisions. In this multicenter, retrospective observational study conducted by the Kinki Breast Cancer Study Group-Translational Research (KBCSG-TR), we aimed to examine real-world effectiveness following T-DM1 discontinuation (post–T-DM1 treatment) in patients with HER2-positive, unresectable and/or metastatic breast cancer.

## Methods

### Study design and patient population

The KBCSG-TR 1917 study (UMIN000038296) was a multicenter, retrospective, observational study conducted in patients with HER2-positive, unresectable and/or metastatic breast cancer. The data cut-off date for all analyses was 31 July 2019. Electronic medical records from five sites in Japan were used to identify patients who had received T-DM1 treatment (either as a single-agent or in a combination therapy regimen) between 1 January 2014 and 31 December 2018.

The inclusion criteria were as follows: women aged ≥20 years at the start of post–T-DM1 treatment; pathological diagnosis of HER2-positive, unresectable and metastatic breast cancer (immunohistochemistry [IHC] 3+, IHC 2+ and in situ hybridization [ISH]+, or IHC not evaluated and ISH+) according to the Japanese Breast Cancer Society “General rules for clinical and pathological recording of breast cancer” [21] at the time of diagnosis; and initiation of at least one line of drug therapy (anti-HER2 targeted therapy, molecular targeted therapy, chemotherapy, or endocrine therapy) for HER2-positive, unresectable and/or metastatic breast cancer between 1 January 2014 and 31 December 2018 immediately after T-DM1 treatment discontinuation. The exclusion criteria were as follows: patient had received an approved or new investigational drug without a breast cancer indication (as defined in Japan) in any clinical trial immediately following T-DM1 treatment discontinuation; or expression (prior to the database lock) of the intention not to participate in this study using the opt-out approach.

We considered that the median PFS in the control group (treatment of the physician’s choice) in the TH3RESA trial was 3.3 months [15], and thus determined that a 4-month observation period for real-world PFS (rwPFS) assessment would be sufficient.

### Ethics approval

This retrospective observational study involving human participants was conducted in accordance with the ethical principles found in the Declaration of Helsinki, the Ethical Guidelines for Medical and Health Research Involving Human Subjects, and in compliance with the study protocol and all applicable local and national ethical guidelines. This study was approved by the ethic screening committee of Osaka Prefectural Hospital Organization Osaka International Cancer Institute. As a non-interventional study with no invasive procedures or human-derived specimens, informed consent was neither required nor obtained from study participants; the opt-out approach was employed to ensure that patients had the opportunity to refuse the registration of their information in this study.

### Patient registration and data collection

Patients with breast cancer who had a history of T-DM1 treatment were identified by study investigators using the medical record search system at each study site. Once extracted, patient records were checked and those who met all of the inclusion criteria and none of the exclusion criteria were considered as study participants. Eligible patients were then consecutively registered from 3 September 2019 to 22 November 2019. Anonymized data from the medical records of all patients registered in the study were entered into the DATATRAK ONE® system (DATATRAK Int., Mayfield Heights, OH, USA).

### Study outcomes

Outcome assessments included rwPFS, time-to-treatment failure (TTF), OS, ORR, and clinical benefit rate (CBR). rwPFS was selected as the outcome measure due to the nature of the study design (using electronic medical records), and was in line with previous, similar, analyses [22].

Tumor responses were assessed by the study investigators in patients with measurable target lesions, ideally complying with the Response Evaluation Criteria in Solid Tumors (RECIST) guidelines (version 1.1). Cancer progression was diagnosed by the attending physician at the time of treatment. In this study, priority was given to the attending physician over the study investigator’s assessment of documented cancer progression based on RECIST (version 1.1). rwPFS was counted from the start date of post–T-DM1 drug therapy to the date of the first documented cancer progression (after the start date of post–T-DM1 drug therapy) or the date of all-cause death, whichever occurred first. The last date of documented rwPFS was the earliest occurring date of the following: post-treatment start date, last visit date, or data cut-off date. TTF was defined as the time from the start date of post–T-DM1 drug therapy to the date of the decision on treatment discontinuation by the attending physician (including disease progression and treatment toxicity). OS was defined as the time from the start date of post–T-DM1 drug therapy to the date of death from any cause. ORR was defined as the percentage of the patient population with the best tumor response (complete response [CR] or partial response [PR]). The CBR was defined as the percentage of the patient population whose best tumor response was CR or PR or who continued treatment for at least 6 months (from the start date of post–T-DM1 drug therapy). Additional details can be found in Additional File 1.

### Statistical analysis

The sample size was set to allow each participating institution to register all eligible patients during the study period. Prior to starting the study, we conducted a survey at each study site and from this were able to estimate the number of patients considered feasible to enroll during the study period. Assuming that five facilities could enroll 20 patients per facility, we expected a total enrolment of 100 patients.

Descriptive statistics were calculated for summaries of patient characteristics. The median survival for rwPFS, TTF, and OS was calculated using the Kaplan–Meier method to estimate the survival curve and the log-rank test to compare the groups; point estimates of survival rates at 6, 12, 18, 24, and 36 months, as well as their 95% confidence intervals (CI) were calculated using Greenwood’s formula. For rwPFS, univariate and multivariate Cox-regression analyses were performed as exploratory analysis; for selection of variables in the multivariate analysis, “previous pertuzumab” and “regimens after T-DM1” were entered using the forced entry method, whereas other variables were selected using the stepwise method. For ORR and CBR, point estimates and 95% CIs were calculated using the Clopper–Pearson method. rwPFS, TTF, OS, ORR, and CBR for subgroup analysis were calculated using the same statistical methods described for the whole population.

Subgroup analyses included study outcome assessments stratified according to Eastern Cooperative Oncology Group Performance Status (ECOG PS; unknown vs 0, ≥ 1 vs 0), central nervous system (CNS) metastases (yes vs no), visceral metastases (yes vs no), hormone receptor status (positive vs negative), HER2 status (IHC3+ vs IHC2+/ISH+ IHC unknown/ISH+), number of treatment lines before T-DM1 (≥ 2 vs < 2), history of pertuzumab treatment (yes vs no), best response to T-DM1 treatment (CR or PR vs others), and regimens after T-DM1 (anti-HER2 therapy vs without anti-HER2 therapy).

Missing values were not imputed. Statistical analyses were performed using SAS software, version 9.4 (SAS Institute Inc., Cary, NC, USA). All tests were two-sided, with *p* < 0.05 considered statistically significant.

## Results

### Study population

**Fig. 1:**
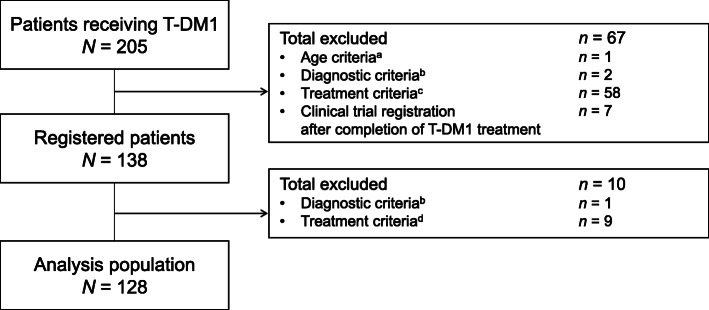
Patient disposition. **A** Aged ≥ 20 years at the start of drug therapy following T-DM1 treatment discontinuation. **B** Not pathologically diagnosed with unresectable and/or metastatic human epidermal growth factor receptor 2 (HER2)-positive breast cancer. **C** Did not start at least one line of drug therapy (anti-HER2 therapy, molecular targeted therapy, chemotherapy, and endocrine therapy) for unresectable and/or metastatic HER2-positive breast cancer following T-DM1 treatment discontinuation between 1 January 2014 and 31 December 2018. **D** At least one line of drug therapy (anti-HER2 therapy, molecular targeted therapy, chemotherapy, and endocrine therapy) was started for unresectable and/or metastatic HER2-positive breast cancer following T-DM1 treatment discontinuation, but the start date was 1 January 2019 or later. *T-DM1* trastuzumab emtansine

Patient disposition is shown in Fig. 1. Briefly, 205 patients who had received T-DM1 within the study period of 1 January 2014 and 31 December 2018 were identified from the medical records search. After evaluation against the inclusion/exclusion criteria, 138 patients were registered. The main reason for exclusion was that the start date of post–T-DM1 treatment did not occur within the study period. Ten registered patients were excluded from the analysis and the main reason for exclusion was that patients did not meet the inclusion criteria after completing T-DM1 treatment. The data collected for the remaining 128 patients were analyzed; the analysis population with measurable lesions included 111 patients.

Table 1 shows the patients’ characteristics as well as those stratified by regimen type following T-DM1 treatment and type of metastatic cancer, and those stratified by recurrent / de novo cancer are shown in Additional File 1. In the total analysis population, the median (range) age was 59.0 (27–84) years. All patients were female with HER2-positive breast cancer (IHC3+, 81% [*n* = 104]), and 65% (*n* = 83) were hormone receptor-positive. Recurrent disease was reported for 64% (*n* = 82) of patients (total analysis population), and 36% (*n* = 46) had de novo disease (defined as Stage IV [Any T + Any N + M1] or recurrence within 6 months of the start of initial treatment). Regarding prior anti-HER2 therapies before T-DM1 (total analysis population), trastuzumab, pertuzumab, and lapatinib had been received by 94% (*n* = 120), 56% (*n* = 72), and 28% (*n* = 36) of patients, respectively. Regarding prior chemotherapy, 84% (*n* = 108) had received taxane-based therapy and 50% (*n* = 64) had received anthracycline-based therapy (Table 1). The median (range) T-DM1 treatment duration was 5.1 (0.0–41.4) months. Forty-six (36%) patients had a best tumor response of CR or PR with T-DM1 treatment. The most common reason for T-DM1 discontinuation was disease progression (80%).

**Table 1:** Patient demographic characteristics according to regimen after T-DM1

	All (***N*** = 128)	Regimen after T-DM1	
		Anti-HER2 therapy (***n*** = 105)	Without anti-HER2 therapy (***n*** = 23)
**Age (years)**			
Median (range)	59.0 (27–84)	60.0 (36–84)	57.0 (27–78)
≥ 60 years	62 (48.4)	54 (51.4)	8 (34.8)
**ECOG PS**			
0	67 (52.3)	57 (54.3)	10 (43.5)
1	25 (19.5)	20 (19.0)	5 (21.7)
≥ 2	9 (7.0)	6 (5.7)	3 (13.0)
Unknown	27 (21.1)	22 (21.0)	5 (21.7)
**Hormone receptor status**			
Positive	83 (64.8)	66 (62.9)	17 (73.9)
Negative	43 (33.6)	38 (36.2)	5 (21.7)
Unknown	2 (1.6)	1 (1.0)	1 (4.3)
**HER2 status**			
IHC3+	104 (81.3)	83 (79.0)	21 (91.3)
IHC2+ and ISH+	21 (16.4)	19 (18.1)	2 (8.7)
IHC not performed and ISH + ^a^	3 (2.3)	3 (2.9)	0 (0.0)
**Type of metastatic breast cancer**			
De novo^b^	46 (35.9)	39 (37.1)	7 (30.4)
Recurrent	82 (64.1)	66 (62.9)	16 (69.6)
Disease-free interval (months), median (range)^c^	39.59 (7.9–198.3)	39.59 (7.9–198.3)	42.00 (9.9–193.8)
**Metastatic stie at initial metastatic diagnosis**			
Liver	42 (32.8)	34 (32.4)	8 (34.8)
Lung	36 (28.1)	33 (31.4)	3 (13.0)
Bone	44 (34.4)	37 (35.2)	7 (30.4)
Peritoneal dissemination	7 (5.5)	6 (5.7)	1 (4.3)
Ascites	0 (0.0)	0 (0.0)	0 (0.0)
CNS	5 (3.9)	5 (4.8)	0 (0.0)
Skin/subcutaneous soft tissues	17 (13.3)	14 (13.3)	3 (13.0)
Lymph nodes	60 (46.9)	50 (47.6)	10 (43.5)
Others	6 (4.7)	5 (4.8)	1 (4.3)
**Drug therapy prior to T-DM1 treatment**			
**Anti-HER2 therapy**			
Trastuzumab	120 (93.8)	99 (94.3)	21 (91.3)
Pertuzumab	72 (56.3)	58 (55.2)	14 (60.9)
Lapatinib	36 (28.1)	28 (26.7)	8 (34.8)
None	6 (4.7)	5 (4.8)	1 (4.3)
**Chemotherapy**			
Anthracycline-based	64 (50.0)	50 (47.6)	14 (60.9)
Taxane-based	108 (84.4)	88 (83.8)	20 (87.0)
Paclitaxel	56 (43.8)	42 (40.0)	14 (60.9)
Docetaxel	80 (62.5)	67 (63.8)	13 (56.5)
Neither anthracycline nor taxane	19 (14.8)	16 (15.2)	3 (13.0)
Capecitabine/S-1	47 (36.7)	34 (32.4)	13 (56.5)
**No. of chemotherapy treatments before T-DM1 in any setting**			
0	13 (10.2)	11 (10.5)	2 (8.7)
1	36 (28.1)	32 (30.5)	4 (17.4)
2	25 (19.5)	21 (20.0)	4 (17.4)
≥ 3	54 (42.2)	41 (39.0)	13 (56.5)
**Duration from initial metastatic diagnosis to the start of T-DM1 treatment (months)**			
Median (range)	22.00 (0.03–174.9)	–	–
**Best response with T-DM1**			
CR, PR	46 (35.9)	42 (40.0)	4 (17.4)
SD, non-CR/non-PD, PD	80 (62.5)	62 (59.0)	18 (78.3)
Unknown	2 (1.6)	1 (1.0)	1 (4.3)
**Duration of T-DM1 treatment (months)**			
Median (range)	5.09 (0.03–41.4)	5.78 (0.7–41.4)	2.33 (0.03–26.5)
< 6 months	74 (57.8)	54 (51.4)	20 (87.0)
≥ 6 to < 12 months	30 (23.4)	29 (27.6)	1 (4.3)
≥ 12 months	24 (18.8)	22 (21.0)	2 (8.7)
**Reason for T-DM1 treatment discontinuation**			
Disease progression	102 (79.7)	82 (78.1)	20 (87.0)
Toxicity	21 (16.4)	19 (18.1)	2 (8.7)
Other	5 (3.9)	4 (3.8)	1 (4.3)
**Metastatic site at start of drug therapy after T-DM1 treatment discontinuation**			
Viscera	89 (69.5)	72 (68.6)	17 (73.9)
Skin/subcutaneous soft tissues/lymph nodes	76 (59.4)	64 (61.0)	12 (52.2)
Bone	53 (41.4)	42 (40.0)	11 (47.8)
CNS	17 (13.3)	15 (14.3)	2 (8.7)
Other	9 (7.0)	8 (7.6)	1 (4.3)

Data are *n* (%) unless otherwise indicated

^a^The study protocol states that “IHC3+ or IHC2+/ISH+ tumors are defined as HER2-positive”. However, at the case review meeting, it was determined that study patients with “IHC not performed and ISH+” who underwent anti-HER2 therapy were to be regarded as HER2-positive

^b^Defined as Stage IV (Any T + Any N + M1) or recurrence within 6 months after the start of initial treatment

^c^A single missing case was excluded from recurrent cases

*CNS* central nervous system, *CR* complete response, *ECOG PS* Eastern Cooperative Oncology Group Performance Status, *HER2* human epidermal growth factor receptor 2, *IHC* immunohistochemistry, *ISH* in situ hybridization, *PD* progressive disease, *PR* partial response, *SD* stable disease, *T-DMI* trastuzumab emtansine

At the start of post–T-DM1 treatment, 67 patients (52%) had an ECOG PS score of 0, 25 patients (20%) had a score of 1, nine patients (7%) had a score of ≥2, and 27 patients (21%) had an unknown score. The metastatic sites at post–T-DM1 treatment initiation (total analysis population) were as follows: visceral (70%, *n* = 89), skin/subcutaneous soft tissue/lymph node (59%, *n* = 76), bone (41%, *n* = 53), and CNS (13%, *n* = 17). Overall, characteristics in the population with measurable lesions were similar to those in the total analysis population.

### Treatment regimens

Treatment regimens after T-DM1 (total analysis population) are shown in Table 2. Among the 128 patients analyzed, 105 (82%) patients received anti-HER2 therapy and 23 (18%) patients received regimens without anti-HER2 therapy. The following treatment regimens were used for patients who received anti-HER2 therapy: pertuzumab-containing therapy (28%) including a combination of pertuzumab with trastuzumab ± chemotherapy or endocrine therapy, trastuzumab-containing therapy (excluding pertuzumab) (27%), and lapatinib + capecitabine therapy (27%).

**Table 2:** Treatment regimen stratified by whether the patient was treated with or without anti-HER2 therapy

	All (***N*** = 128)
**Anti-HER2 therapy**	105 (82.0)
Trastuzumab + pertuzumab + chemotherapy	32 (25.0)
Trastuzumab + pertuzumab + endocrine therapy	1 (0.8)
Trastuzumab + pertuzumab	3 (2.3)
Trastuzumab + chemotherapy	16 (12.5)
Trastuzumab + endocrine therapy	9 (7.0)
Trastuzumab alone	10 (7.8)
Lapatinib + capecitabine	34 (26.6)
**Regimens without anti-HER2 therapy**	23 (18.0)
Bevacizumab + paclitaxel	12 (9.4)
Other chemotherapy	5 (3.9)
Everolimus + exemestane	2 (1.6)
Endocrine alone	4 (3.1)

Data are *n* (%)

*HER2* human epidermal growth factor receptor 2

### Efficacy outcomes

**Fig. 2:**
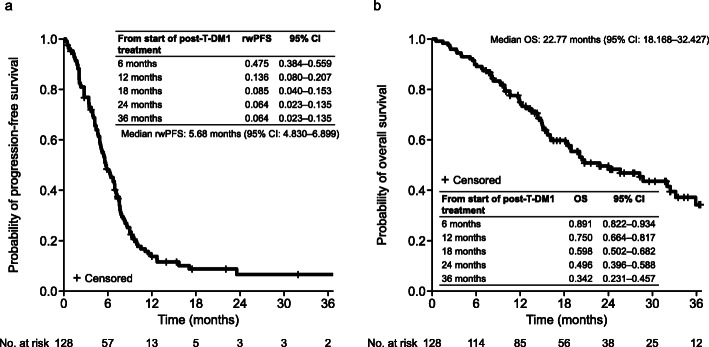
Product-limit survival estimates for (**A**) real-world progression-free survival and (**B**) overall survival. *CI* confidence interval, *OS* overall survival, *rwPFS* real-world progression-free survival

The median (range) follow-up time was 15.5 (0.5–57.4) months. The median rwPFS (95% CI; number of events) was 5.7 months (4.8–6.9; 109) (Fig. 2A) and the median TTF (95% CI; number of events) was 5.6 months (4.6–6.4; 117). The median OS (95% CI; number of events) was 22.8 months (18.2–32.4; 65) (Fig. 2B). Among the 111 patients with measurable lesions, one patient (0.9%) achieved CR and 24 (22%) achieved PR, with an ORR of 23% (25/111; 95% CI: 15.1–31.4). SD was achieved in 34 patients (31%), 44 had PD (40%), and eight (7%) had an unknown response. The CBR (95% CI) was 48% (38.8–56.7).

### Subgroup analysis

Subgroup analysis revealed that rwPFS was longer (per the 95% CI) in patients with the following characteristics: an ECOG PS score of 0 (6.7 months [95% CI: 5.4–7.4]) vs an ECOG PS score of ≥1 (3.9 months [95% CI: 2.1–5.8]), HER2 IHC 3+ (6.2 months [95% CI: 5.1–7.1]) vs IHC 2+/ISH+ and IHC unknown/ISH+ (3.9 months [95% CI: 2.1–6.7]), no history of pertuzumab treatment (7.1 months [95% CI: 5.7–7.9]) vs history of pertuzumab treatment (4.9 months [95% CI: 4.0–5.8]), and anti-HER2 therapy (6.3 months [95% CI: 5.1–7.2]) vs regimens without anti-HER2 therapy (4.8 months [95% CI: 1.9–5.9]). Other subgroup outcomes of TTF, OS, ORR, and CBR are shown in Additional File 1.

### Exploratory analysis of clinical factors associated with rwPFS

**Fig. 3:**
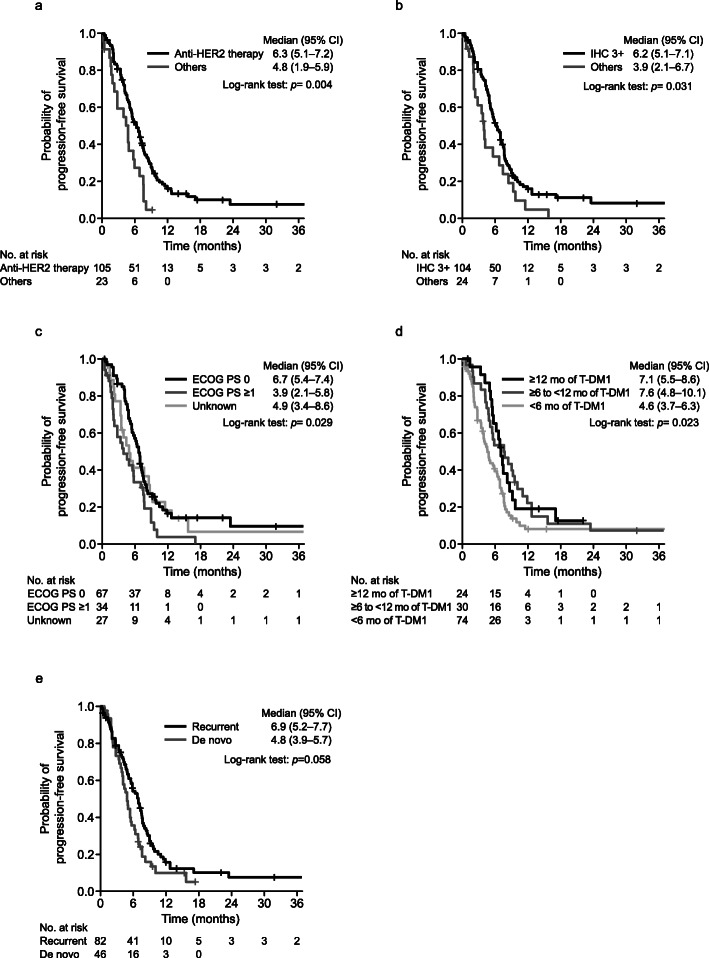
Product-limit survival estimates for real-world progression-free survival in each subgroup; (**A**) anti-HER2 therapy vs others, (**B**) HER2 IHC3+ vs others, (**C**) ECOG PS 0 vs ≥ 1 vs unknown, (**D**) ≥ 12 months duration of T-DM1 vs 6–12 months vs < 6 months, and (**E**) recurrent vs de novo. *CI* confidence interval, *ECOG PS* Eastern Cooperative Oncology Group Performance Status, *HER2* human epidermal growth factor receptor 2, *IHC* immunohistochemistry, *T-DMI* trastuzumab emtansine

To determine clinical variables associated with better median rwPFS, univariate and multivariate Cox-regression analyses were performed. An ECOG PS score of 0 (≥1 vs 0: hazard ratio 1.81, 95% CI; 1.16–2.84), recurrent type (HR = 0.68, 95% CI; 0.42–0.97), a HER2 immunohistochemistry score of 3+ (HR = 0.52, 95% CI; 0.31–0.86), more than 12 months duration of T-DM1 treatment (≥ 12 months vs < 6 months: HR = 0.55, 95% CI; 0.32–0.96), and anti-HER2 therapy (HR = 0.48, 95% CI; 0.28–0.83) were identified as independent variables for rwPFS in both analyses (Table 3). Kaplan–Meier curves for each subgroup are shown in Fig. 3A–E. The median rwPFS for anti-HER2 therapy was significantly better than that of regimens without anti-HER2 therapy (*p* = 0.004, Fig. 3A). Interestingly and unexpectedly, rwPFS tended to be shorter in the de novo type than recurrent type (*p* = 0.058) (Fig. 3E).

**Table 3:** Univariate/multivariate Cox-regression analysis for real-world progression-free survival

	Univariate (***N*** = 128)		Multivariate^**a**^ (***N*** = 126)			
	HR^**b**^	95% CI	***p*** value	HR^**b**^	95% CI	***p*** value
Age (years)						
≥ 60 vs < 60	0.70	0.478–1.024	0.066	–	–	–
ECOG PS						
Unknown vs 0	1.22	0.748–1.991	0.425	0.95	0.555–1.621	0.848
≥ 1 vs 0	1.81	1.161–2.824	0.009	1.81	1.158–2.843	0.009
Recurrent / de novo, recurrent vs de novo	0.68	0.461–1.017	0.061	0.64	0.423–0.968	0.034
CNS metastasis, yes vs no	1.02	0.577–1.790	0.956	–	–	–
Visceral metastasis, yes vs no	1.08	0.715–1.623	0.724	–	–	–
Hormone receptor status, positive vs negative	0.97	0.650–1.449	0.883	–	–	–
HER2 status, IHC 3+ vs IHC 2+/ISH + or IHC unknown/ISH+	0.60	0.374–0.961	0.034	0.52	0.307–0.864	0.012
Number of treatment lines before T-DM1 treatment, ≥ 2 vs < 2	1.01	0.688–1.491	0.949	–	–	–
History of pertuzumab treatment, yes vs no	1.55	1.054–2.286	0.026	1.28	0.855–1.903	0.232
History of lapatinib treatment, yes vs no	0.94	0.620–1.439	0.789	–	–	–
Regimen with anthracyclines and/or taxanes before T-DM1						
Yes (both) vs no (both)	1.38	0.784–2.421	0.265	–	–	–
Yes (either one) vs no (both)	1.57	0.876–2.810	0.130	–	–	–
Best response during T-DM1 treatment						
CR or PR vs SD, non-CR/non-PD, PD, or unknown	0.70	0.468–1.040	0.077	–	–	–
Duration of T-DM1 treatment						
≥ 12 months vs < 6 months	0.57	0.339–0.959	0.034	0.55	0.318–0.959	0.035
6–12 months vs < 6 months	0.60	0.377–0.940	0.026	0.65	0.401–1.064	0.087
Duration from the last day of T-DM1 to the start of the next regimen						
≥ 2 months vs < 1 month	0.71	0.405–1.245	0.233	–	–	–
1–2 months vs < 1 month	1.28	0.843–1.942	0.247	–	–	–
Regimens after T-DM1						
Anti-HER2 therapy vs without anti-HER2 therapy	0.50	0.305–0.814	0.005	0.48	0.282–0.826	0.008

^a^Stepwise method was applied after forcibly inserting “History of pertuzumab treatment” and “regimens after T-DM1”

^b^HR with reference to the second comparator

*CNS* central nervous system, *CI* confidence interval, *CR* complete response, *ECOG PS* Eastern Cooperative Oncology Group Performance Status, *HER2* human epidermal growth factor receptor 2, *HR* hazard ratio, *IHC* immunohistochemistry, *ISH* in situ hybridization, *PD* progressive disease, *PR* partial response, *SD* stable disease, *T-DM1* trastuzumab emtansine

## Discussion

In this multicenter, retrospective, observational study, we found that among 128 patients with HER2-positive metastatic breast cancer in real-world clinical practice, 82% were treated with post–T-DM1 regimens that included anti-HER2 therapy and 18% were treated with post–T-DM1 regimens without anti-HER2 therapy. We report a median rwPFS and OS for post–T-DM1 therapy of 5.7 and 22.8 months, respectively; patients with measurable lesions had an ORR of 23%. Both the PFS and ORR were similar to the data previously reported in the control arms of several recent global clinical trials, including the NALA, SOPHIA, monarcHER, and HER2CLIMB studies [23–27].

In the present study, a subgroup analysis revealed that treatment regimens including anti-HER2 therapy achieved better outcomes than those that did not include anti-HER2 therapy. Several previous studies have reported a benefit for trastuzumab beyond progression strategy [28, 29]. A report from Germany of patients with HER2-positive early and advanced breast cancer found that continuing trastuzumab treatment, combined with capecitabine beyond progression, significantly improved ORR and PFS compared with capecitabine treatment alone, which supports the continued use of anti-HER2 therapy [28]. Additionally, the findings from a retrospective review of patients who received trastuzumab for HER2-positive metastatic breast cancer showed that PFS on first-line trastuzumab-based therapy was a clinically relevant predictive factor for OS when patients were treated with trastuzumab after progression [29]. A retrospective study conducted in the US [30] investigated the clinical outcomes of lapatinib treatment in patients who had been treated with pertuzumab + trastuzumab and/or T-DM1; those patients had a TTF of 6.0 months, which is comparable with the rwPFS reported in our study. In the most recent Clinical Practice Guidelines for systemic treatment of breast cancer (Japanese Breast Cancer Society; 2018 edition [31]), continuation of anti-HER2 therapy is recommended as third-line or later in metastatic settings. The results of our study suggest that continuation of anti-HER2 therapy is an important option after T-DM1 treatment; therefore, this study supports the recent clinical guidelines from the Japanese Breast Cancer Society [31].

This study revealed that even among patients with HER2-positive breast cancer in Japan, some patients receive drug regimens without anti-HER2 therapy. For patients who had discontinued T-DM1 treatment after less than 6 months, there was a tendency to select a regimen without any anti-HER2 therapy, which is associated with shorter rwPFS post–T-DM1. Unlike in the West [32, 33], bevacizumab is approved in Japan for inoperable or recurrent breast cancer [34]. Bevacizumab + paclitaxel was the most frequently prescribed treatment (12/23 patients) as chemotherapy in combination with bevacizumab, which can be used not only as first-line in Japan but also as second-line or greater chemotherapy [31]. Among the available treatment options in Japan, this suggests that physicians are selecting bevacizumab + paclitaxel for patients with tumors that have low sensitivity to anti-HER2 therapy, based on the evidence from studies conducted in patients with HER2-negative tumors [35].

Our study showed better rwPFS in patients who had recurrent vs de novo cancer. Possible reasons for the high malignancy of de novo tumors are as follows: (1) the proportion of patients with liver metastasis, which is considered high risk at the time of initial metastasis diagnosis, was high; and (2) a high proportion of patients had brain metastasis at the time that post–T-DM1 treatment was initiated. Our study did not collect data related to tumor size or other factors, so further exploration of this is needed.

An ECOG PS of 0 (vs ≥ 1) and tumors that were IHC3+ for HER2 (vs IHC2+/ISH+ and IHC unknown/ISH+) have been reported as prognostic factors [36, 37]. It is known that ECOG PS is related to the continuation of treatment and that high HER2 expression is related to sensitivity to anti-HER2 therapy. In the comparator group of our study, efficacy was still insufficient (median rwPFS: 3.9 months) and new treatment options are needed. The HER2CLIMB study recently reported that the addition of tucatinib to trastuzumab and capecitabine improved both PFS and OS in heavily pretreated patients with HER2-positive breast cancer, including those with CNS metastases [26]. Data from the DESTINY-Breast01 study showed that trastuzumab deruxtecan provided sustained antitumor activity in a population of patients with heavily pretreated HER2-positive metastatic breast cancer [38]. The PRECIOUS trial (NCT02514681) is currently being conducted by the Japan Breast Cancer Research Group to evaluate the efficacy of pertuzumab re-treatment in patients with HER2-positive metastatic breast cancer previously treated with pertuzumab + trastuzumab + chemotherapy [39]. Therefore, to improve patient outcomes and prolong survival, clinicians must continue to evaluate new treatments and expand their knowledge of how treatment sequencing may impact subsequent efficacy.

This study had several limitations. As with any retrospective observational study, we relied on accurate record-keeping from treating physicians. Additionally, the sample size was limited and the study only included data from Japanese patients at five study sites; therefore, the results may not be entirely representative of the general population, potentially limiting their generalizability. However, as mentioned above, these data were collected at core cancer treatment hospitals. The data presented herein are specific to the real-world setting in Japan and these findings should be interpreted carefully in the case of real-world settings in other countries. Larger studies would be useful to confirm these findings and to expand on the subgroup analyses.

## Conclusion

We conclude that the results of this study represent the real-world treatment patterns and outcomes of post–T-DM1 therapy in Japan for patients with unresectable and/or metastatic HER2-positive breast cancer. Our results showed that continuation of anti-HER2 treatment and higher expression of HER2 were important factors for longer rwPFS, even in later lines of therapy for patients with HER2-positive breast cancer. However novel therapeutic options are still needed to further improve both PFS and OS in the real world. Future studies clarifying the real-world treatment situation for new anti-HER2 therapies for HER2-positive breast cancer are expected.

## Supplementary Information

**Additional file 1: Supplementary Text 1.** Study outcomes. **Supplementary Table 1**. Patient demographic characteristics (total analysis population and according to recurrent or de novo type). **Supplementary Table 2**. Analysis of outcomes according to subgroup.

## Data Availability

The datasets used and/or analyzed during the current study are available from the corresponding author upon reasonable request.

## Abbreviations

CBR: Clinical benefit rate
CI: Confidence interval
CR: Complete response
ECOG PS: Eastern Cooperative Oncology Group Performance Status
ER: Estrogen receptor
HER2: Human epidermal growth factor receptor 2
HR: Hazard ratio
IHC: Immunohistochemistry
ISH: In situ hybridization
KBCSG-TR: Kinki Breast Cancer Study Group-Translational Research
ORR: Objective response rate
OS: Overall survival
PD: Progressive disease
PFS: Progression-free survival
PR: Partial response
RECIST: Response Evaluation Criteria in Solid Tumors
RR: Response rate
rwPFS: Real-world progression-free survival
SD: Stable disease
T-DM1: Trastuzumab emtansine
TTF: Time-to-treatment failure

## References

[1] Onitilo AA, Engel JM, Greenlee RT, Mukesh BN (2009). Breast cancer subtypes based on ER/PR and Her2 expression: comparison of clinicopathologic features and survival. Clin Med Res.

[2] Gonzalez-Angulo AM, Litton JK, Broglio KR, Meric-Bernstam F, Rakkhit R, Cardoso F, et al. High risk of recurrence for patients with breast cancer who have human epidermal growth factor receptor 2-positive, node-negative tumors 1 cm or smaller. J Clin Oncol. 2009;27(34):5700–6. 10.1200/JCO.2009.23.2025. 10.1200/JCO.2009.23.2025 PMC2792998 19884543

[3] Slamon DJ, Clark GM, Wong SG, Levin WJ, Ullrich A, McGuire WL (1987). Human breast cancer: correlation of relapse and survival with amplification of the HER-2/neu oncogene. Science..

[4] Slamon DJ, Godolphin W, Jones LA, Holt JA, Wong SG, Keith DE, et al. Studies of the HER-2/neu proto-oncogene in human breast and ovarian cancer. Science. 1989;244(4905):707–12. 10.1126/science.2470152. 10.1126/science.2470152 2470152

[5] Harbeck N (2018). Advances in targeting HER2-positive breast cancer. Curr Opin Obstet Gynecol.

[6] Menard S, Fortis S, Castiglioni F, Agresti R, Balsari A (2001). HER2 as a prognostic factor in breast cancer. Oncology..

[7] Slamon DJ, Leyland-Jones B, Shak S, Fuchs H, Paton V, Bajamonde A, et al. Use of chemotherapy plus a monoclonal antibody against HER2 for metastatic breast cancer that overexpresses HER2. N Engl J Med. 2001;344(11):783–92. 10.1056/NEJM200103153441101. 10.1056/NEJM200103153441101 11248153

[8] Maximiano S, Magalhaes P, Guerreiro MP, Morgado M (2016). Trastuzumab in the treatment of breast cancer. BioDrugs..

[9] Cameron D, Casey M, Press M, Lindquist D, Pienkowski T, Romieu CG, et al. A phase III randomized comparison of lapatinib plus capecitabine versus capecitabine alone in women with advanced breast cancer that has progressed on trastuzumab: updated efficacy and biomarker analyses. Breast Cancer Res Treat. 2008;112(3):533–43. 10.1007/s10549-007-9885-0 18188694

[10] Geyer CE, Forster J, Lindquist D, Chan S, Romieu CG, Pienkowski T, et al. Lapatinib plus capecitabine for HER2-positive advanced breast cancer. N Engl J Med. 2006;355(26):2733–43. 10.1056/NEJMoa064320. 10.1056/NEJMoa064320 17192538

[11] Baselga J, Cortes J, Kim SB, Im SA, Hegg R, Im YH, et al. Pertuzumab plus trastuzumab plus docetaxel for metastatic breast cancer. N Engl J Med. 2012;366(2):109–19. 10.1056/NEJMoa1113216. 10.1056/NEJMoa1113216 PMC5705202 22149875

[12] Swain SM, Baselga J, Kim SB, Ro J, Semiglazov V, Campone M, et al. Pertuzumab, trastuzumab, and docetaxel in HER2-positive metastatic breast cancer. N Engl J Med. 2015;372(8):724–34. 10.1056/NEJMoa1413513. 10.1056/NEJMoa1413513 PMC5584549 25693012

[13] Verma S, Miles D, Gianni L, Krop IE, Welslau M, Baselga J, et al. Trastuzumab emtansine for HER2-positive advanced breast cancer. N Engl J Med. 2012;367(19):1783–91. 10.1056/NEJMoa1209124. 10.1056/NEJMoa1209124 PMC5125250 23020162

[14] Krop IE, Kim SB, Martin AG, LoRusso PM, Ferrero JM, Badovinac-Crnjevic T, et al. Trastuzumab emtansine versus treatment of physician's choice in patients with previously treated HER2-positive metastatic breast cancer (TH3RESA): final overall survival results from a randomised open-label phase 3 trial. Lancet Oncol. 2017;18(6):743–54. 10.1016/S1470-2045(17)30313-3. 10.1016/S1470-2045(17)30313-3 28526538

[15] Krop IE, Kim SB, Gonzalez-Martin A, LoRusso PM, Ferrero JM, Smitt M, et al. Trastuzumab emtansine versus treatment of physician's choice for pretreated HER2-positive advanced breast cancer (TH3RESA): a randomised, open-label, phase 3 trial. Lancet Oncol. 2014;15(7):689–99. 10.1016/S1470-2045(14)70178-0. 10.1016/S1470-2045(14)70178-0 24793816

[16] Dieras V, Miles D, Verma S, Pegram M, Welslau M, Baselga J, et al. Trastuzumab emtansine versus capecitabine plus lapatinib in patients with previously treated HER2-positive advanced breast cancer (EMILIA): a descriptive analysis of final overall survival results from a randomised, open-label, phase 3 trial. Lancet Oncol. 2017;18(6):732–42. 10.1016/S1470-2045(17)30312-1. 10.1016/S1470-2045(17)30312-1 PMC5531181 28526536

[17] Kagiyama N, Matsue Y (2018). The time-to-treatment concept in acute heart failure: lessons and implications from REALITY-AHF. Anatol J Cardiol.

[18] NCCN Clinical Practice Guidelines in Oncology: Breast Cancer. https://www.nccn.org/professionals/physician_gls/pdf/breast.pdf.

[19] Hunter FW, Barker HR, Lipert B, Rothe F, Gebhart G, Piccart-Gebhart MJ, et al. Mechanisms of resistance to trastuzumab emtansine (T-DM1) in HER2-positive breast cancer. Br J Cancer. 2020;122(5):603–12. 10.1038/s41416-019-0635-y. 10.1038/s41416-019-0635-y PMC7054312 31839676

[20] Yoshinami T, Hasegawa A, Fujisawa F, Kittaka N, Ishitobi M, Sugimoto N, et al. Abstract P4-22-24: a retrospective study about re-biopsy at disease progression on T-DM1. Cancer Res. 2017;77(4 Suppl):4 -22-24-P24-22-24.

[21] Sakamoto G, Inaji H, Akiyama F, Haga S, Hiraoka M, Inai K, et al. General rules for clinical and pathological recording of breast cancer 2005. Breast Cancer. 2005;12(Suppl):S1–27. 16410755

[22] Griffith SD, Tucker M, Bowser B, Calkins G, Chang CJ, Guardino E, et al. Generating real-world tumor burden endpoints from electronic health record data: comparison of RECIST, radiology-anchored, and clinician-anchored approaches for abstracting real-world progression in non-small cell lung cancer. Adv Ther. 2019;36(8):2122–36. 10.1007/s12325-019-00970-1. 10.1007/s12325-019-00970-1 PMC6822856 31140124

[23] Saura C, Oliveira M, Feng Y-H, Dai M-S, Hurvitz SA, Kim S-B, et al. Neratinib + capecitabine versus lapatinib + capecitabine in patients with HER2+ metastatic breast cancer previously treated with ≥ 2 HER2-directed regimens: Findings from the multinational, randomized, phase III NALA trial. J Clin Oncol. 2019;37(15_suppl):1002.

[24] Rugo HS, Im S-A, Wright GLS, Escriva-de-Romani S, DeLaurentiis M, Cortes J, et al. SOPHIA primary analysis: A phase 3 (P3) study of margetuximab (M) + chemotherapy (C) versus trastuzumab (T) + C in patients (pts) with HER2+ metastatic (met) breast cancer (MBC) after prior anti-HER2 therapies (Tx). J Clin Oncol. 2019;37(15_suppl):1000.

[25] Tolaney SM, Bourayou N, Goel S, Forrester T, André F (2016). monarcHER: A phase 2 randomized open-label study of abemaciclib plus trastuzumab (T) with or without fulvestrant (F) compared to standard-of-care chemotherapy of physician's choice plus T in women with HR +, HER2+ advanced breast cancer. Ann Oncol.

[26] Murthy RK, Loi S, Okines A, Paplomata E, Hamilton E, Hurvitz SA, et al. Tucatinib, trastuzumab, and capecitabine for HER2-positive metastatic breast cancer. N Engl J Med. 2020;382(7):597–609. 10.1056/NEJMoa1914609. 10.1056/NEJMoa1914609 31825569

[27] A study of neratinib plus capecitabine versus lapatinib plus capecitabine in patients with HER2+ metastatic breast cancer who have received two or more prior HER2 directed regimens in the metastatic setting (NALA). https://clinicaltrials.gov/ct2/show/results/NCT01808573?view=results. Accessed 8 March 2020.

[28] von Minckwitz G, du Bois A, Schmidt M, Maass N, Cufer T, de Jongh FE, et al. Trastuzumab beyond progression in human epidermal growth factor receptor 2-positive advanced breast cancer: a german breast group 26/breast international group 03-05 study. J Clin Oncol. 2009;27(12):1999–2006. 10.1200/JCO.2008.19.6618. 10.1200/JCO.2008.19.6618 19289619

[29] Rayson D, Lutes S, Walsh G, Sellon M, Colwell B, Dorreen M, et al. Trastuzumab beyond progression for HER2 positive metastatic breast cancer: progression-free survival on first-line therapy predicts overall survival impact. Breast J. 2014;20(4):408–13. 10.1111/tbj.12284. 10.1111/tbj.12284 24985529

[30] Baez-Vallecillo L, Raghavendra AS, Hess KR, Barcenas CH, Moulder SL, Tripathy D, et al. Lapatinib activity in metastatic human epidermal growth factor receptor 2-positive breast cancers that received prior therapy with trastuzumab, pertuzumab, and/or ado-trastuzumab emtansine (T-DM1). Breast Cancer Res Treat. 2019;176(1):227–34. 10.1007/s10549-018-05081-z. 10.1007/s10549-018-05081-z PMC12831230 30977027

[31] Shimoi T, Nagai SE, Yoshinami T, Takahashi M, Arioka H, Ishihara M, et al. The Japanese breast Cancer society clinical practice guidelines for systemic treatment of breast cancer, 2018 edition. Breast Cancer. 2020;27(3):322–31. 10.1007/s12282-020-01085-0. 10.1007/s12282-020-01085-0 PMC8062371 32240526

[32] AVASTIN (bevacizumab) [prescribing information] https://www.accessdata.fda.gov/drugsatfda_docs/label/2014/125085s301lbl.pdf. .

[33] Union Register of medicinal products for human use. Product information: Avastin. https://ec.europa.eu/health/documents/community-register/html/h300.htm. Accessed 11 March 2021.

[34] Avastin (bevacizumab) [package insert]. https://www.info.pmda.go.jp/go/pack/4291413A1022_1_23/. Accessed 11 March 2021. [In Japanese].

[35] Wang X, Huang C, Li M, Gu Y, Cui Y, Li Y (2014). The efficacy of bevacizumab plus paclitaxel as first-line treatment for HER2-negative metastatic breast cancer: a meta-analysis of randomized controlled trials. Tumour Biol.

[36] Gamucci T, Pizzuti L, Natoli C, Mentuccia L, Sperduti I, Barba M, et al. A multicenter REtrospective observational study of first-line treatment with PERtuzumab, trastuzumab and taxanes for advanced HER2 positive breast cancer patients. RePer Study. Cancer Biol Ther. 2019;20(2):192–200. 10.1080/15384047.2018.1523095 PMC6343690 30403909

[37] Vici P, Pizzuti L, Michelotti A, Sperduti I, Natoli C, Mentuccia L, et al. A retrospective multicentric observational study of trastuzumab emtansine in HER2 positive metastatic breast cancer: a real-world experience. Oncotarget. 2017;8(34):56921–31. 10.18632/oncotarget.18176 PMC5593613 28915642

[38] Modi S, Saura C, Yamashita T, Park YH, Kim SB, Tamura K, et al. Trastuzumab deruxtecan in previously treated HER2-positive breast cancer. N Engl J Med. 2020;382(7):610–21. 10.1056/NEJMoa1914510. 10.1056/NEJMoa1914510 PMC7458671 31825192

[39] Yamamoto Y, Iwata H, Ueno T, Taira N, Kashiwaba M, Takahashi M, et al. A randomized, open-label, phase III trial of pertuzumab retreatment in HER2-positive locally advanced/metastatic breast cancer patients previously treated with pertuzumab, trastuzumab and chemotherapy: the Japan breast Cancer research group-M05 PRECIOUS study. Jpn J Clin Oncol. 2018;48(9):855–9. 10.1093/jjco/hyy097. 10.1093/jjco/hyy097 30020510

