# Supplementary material for: Real-world effectiveness of post-trastuzumab emtansine treatment in patients with HER2-positive, unresectable and/or metastatic breast cancer: a retrospective observational study (KBCSG-TR 1917)
Source: BMC Cancer. 2021 Jul 9;21:795. doi: 10.1186/s12885-021-08504-1 (PMC8268506; doi:10.1186/s12885-021-08504-1)
Supplement: Supplementary file 1 — Additional file 1: Supplementary Text 1. Study outcomes. Supplementary Table 1. Patient demographic characteristics (total analysis population and according to recurrent or de novo type). Supplementary Table 2. Analysis of outcomes according to subgroup. [file 12885_2021_8504_MOESM1_ESM.docx]

**Supplementary information**

**Real-world effectiveness of post-trastuzumab emtansine treatment in patients with HER2-positive, unresectable and/or metastatic breast cancer: A retrospective observational study (KBCSG-TR 1917)**

Takahiro Nakayama^1^, Tetsuhiro Yoshinami^2^, Hiroyuki Yasojima^3^, Nobuyoshi Kittaka^1^, Masato Takahashi^4^, Shoichiro Ohtani^5^, Seung Jin Kim^2^, Hiroyuki Kurakami^6^, Naoko Yamamoto^6^, Tomomi Yamada^6^, Takehiko Takata^7^, Norikazu Masuda^3^

^1^ Department of Breast and Endocrine Surgery, Osaka International Cancer Institute, Osaka, Japan

3-1-69, Otemae, Chuo-ku, Osaka, Osaka 541-8567, Japan

^2^ Department of Breast and Endocrine Surgery, Osaka University Hospital, Osaka, Japan

2-15, Yamadaoka, Suita, Osaka 565-0871, Japan

^3^ Department of Surgery, Breast Oncology, National Hospital Organization Osaka National Hospital, Osaka, Japan

2-1-14, Hoenzaka, Chuo-ku, Osaka, Osaka 540-0006, Japan

^4^ Department of Breast Surgery, National Hospital Organization Hokkaido Cancer Center, Hokkaido, Japan

2-3-54, Kikusui 4-jo Shiroishi-ku, Sapporo, Hokkaido, 003-0804, Japan

^5^ Department of Breast Surgery, Hiroshima City Hiroshima Citizens Hospital, Hiroshima, Japan

7-33 Motomachi, Naka-ku, Hiroshima, Hiroshima, 730-8518, Japan

^6^ Department of Medical Innovation, Osaka University Hospital, Osaka, Japan

2-15, Yamadaoka, Suita, Osaka 565-0871, Japan

^7^ Oncology Medical Science Department, Daiichi Sankyo Co., Ltd., Tokyo, Japan

3-5-1, Nihonbashi-honcho, Chuo-ku, Tokyo 103-8426, Japan

**Corresponding author:**

Takahiro Nakayama

Department of Breast and Endocrine Surgery, Osaka International Cancer Institute, Osaka, Japan

3-1-69, Otemae, Chuo-ku, Osaka, Osaka 541-8567, Japan

Tel: +81-6-6897-5037

E-mail: taqnakayama@gmail.com

ORCID ID: 0000-0002-3713-1624

**Supplementary Text 1** Study outcomes

rwPFS was counted from the start date of post–T-DM1 drug therapy. The event occurrence date was defined as the date of the first documented disease progression (after the start date of post–T-DM1 drug therapy) or the date of all-cause death, whichever occurred first. The last date of documented rwPFS was the earliest occurring date of the following: post-treatment start date, last visit date, or 31 July 2019.

TTF was defined as the time from the start date of post–T-DM1 drug therapy to the date of the decision on treatment discontinuation by the attending physician (including disease progression and treatment toxicity). However, if the decision date was unknown, TTF was defined as the next scheduled administration date after the last administration day or the date of all-cause death, whichever occurred first. The next scheduled administration date was considered either the scheduled administration date for the next treatment cycle after the last administration day documented for the post–T-DM1 drug therapy, or the next scheduled administration date if it was within the same treatment cycle. If no event occurred, the last confirmation date of treatment success was considered to be the earliest date among the following: start date, last visit date, or 31 July 2019.

OS was defined as the time from the start date of post–T-DM1 drug therapy to the date of death from any cause. If no death was reported before the data cut-off date, OS was censored at the last visit date at which the patient was known to be alive or the data cut-off date (31 July 2019).

ORR was defined as the percentage of the patient population with the best tumor response (CR or PR); tumor response was assessed using RECIST (version 1.1). The ORR was calculated for the population who had measurable lesions (as determined by the investigator).

The CBR was defined as the percentage of the patient population whose best tumor response was CR or PR or who continued treatment for at least 6 months (from the start date of post–T-DM1 drug therapy).

**Supplementary Table 1** Patient demographic characteristics (total analysis population and according to recurrent or de novo type)

|  | **All**  **(*N* = 128)** | **Recurrent / de novo** | |
| --- | --- | --- | --- |
|  |  | **Recurrent**  **(*n* = 82)** | **De novo stage IV**  **(*n* = 46)** |
| **Age (years)** | | | |
| Median (range) | 59.0 (27–84) | 60.5 (36–82) | 58.0 (27–84) |
| ≥ 60 years | 62 (48.4) | 43 (52.4) | 19 (41.3) |
| **ECOG PS** | | | |
| 0 | 67 (52.3) | 43 (52.4) | 24 (52.2) |
| 1 | 25 (19.5) | 16 (19.5) | 9 (19.6) |
| ≥ 2 | 9 (7.0) | 6 (7.3) | 3 (6.5) |
| Unknown | 27 (21.1) | 17 (20.7) | 10 (21.7) |
| **Hormone receptor status** | | | |
| Positive | 83 (64.8) | 54 (65.9) | 29 (63.0) |
| Negative | 43 (33.6) | 26 (31.7) | 17 (37.0) |
| Unknown | 2 (1.6) | 2 (2.4) | 0 (0.0) |
| **HER2 status** | | | |
| IHC3+ | 104 (81.3) | 68 (82.9) | 36 (78.3) |
| IHC2+ and ISH+ | 21 (16.4) | 12 (14.6) | 9 (19.6) |
| IHC not performed and ISH+^a^ | 3 (2.3) | 2 (2.4) | 1 (2.2) |
| **Type of metastatic breast cancer** | | | |
| De novo^b^ | 46 (35.9) | - | 46 (100) |
| Recurrent | 82 (64.1) | 82 (100) | - |
| Disease-free interval (months), median (range)^c^ | 39.59 (7.9–198.3) | 39.59 (7.9–198.3) | - |
| **Metastatic site at initial metastatic diagnosis** | | | |
| Liver | 42 (32.8) | 21 (25.6) | 21 (45.7) |
| Lung | 36 (28.1) | 26 (31.7) | 10 (21.7) |
| Bone | 44 (34.4) | 18 (22.0) | 26 (56.5) |
| Peritoneal dissemination | 7 (5.5) | 3 (3.7) | 4 (8.7) |
| Ascites | 0 (0.0) | 0 (0.0) | 0 (0.0) |
| CNS | 5 (3.9) | 4 (4.9) | 1 (2.2) |
| Skin/subcutaneous soft tissues | 17 (13.3) | 9 (11.0) | 8 (17.4) |
| Lymph nodes | 60 (46.9) | 30 (36.6) | 30 (65.2) |
| Others | 6 (4.7) | 4 (4.9) | 2 (4.3) |
| **Drug therapy prior to T-DM1 treatment** | | | |
| **Anti-HER2 therapy** | | | |
| Trastuzumab | 120 (93.8) | 80 (97.6) | 40 (87.0) |
| Pertuzumab | 72 (56.3) | 42 (51.2) | 30 (65.2) |
| Lapatinib | 36 (28.1) | 27 (32.9) | 9 (19.6) |
| None | 6 (4.7) | 0 (0.0) | 6 (13.0) |
| **Chemotherapy** | | | |
| Anthracycline-based | 64 (50.0) | 54 (65.9) | 10 (21.7) |
| Taxane-based | 108 (84.4) | 69 (84.1) | 39 (84.8) |
| Paclitaxel | 56 (43.8) | 43 (52.4) | 13 (28.3) |
| Docetaxel | 80 (62.5) | 49 (59.8) | 31 (67.4) |
| Neither anthracycline nor taxane | 19 (14.8) | 12 (14.6) | 7 (15.2) |
| Capecitabine/S-1 | 47 (36.7) | 37 (45.1) | 10 (21.7) |
| **No. of chemotherapy treatments before T-DM1 in any setting** | | | |
| 0 | 13 (10.2) | 6 (7.3) | 7 (15.2) |
| 1 | 36 (28.1) | 10 (12.2) | 26 (56.5) |
| 2 | 25 (19.5) | 21 (25.6) | 4 (8.7) |
| ≥ 3 | 54 (42.2) | 45 (54.9) | 9 (19.6) |
| **Duration from initial metastatic diagnosis to the start of T-DM1 treatment (months)** | | | |
| Median (range) | 22.00 (0.03–174.9) | 29.93 (0.03–174.9) | 15.59 (0.3–122.3) |
| **Best response with T-DM1** | |  |  |
| CR, PR | 46 (35.9) | 24 (29.3) | 22 (47.8) |
| SD, non-CR/non-PD, PD | 80 (62.5) | 56 (68.3) | 24 (52.2) |
| Unknown | 2 (1.6) | 2 (2.4) | 0 (0.0) |
| **Duration of T-DM1 treatment (months)** | | | |
| Median (range) | 5.09 (0.03–41.4) | 5.03 (0.03–41.4) | 6.51 (0.7–26.5) |
| < 6 months | 74 (57.8) | 52 (63.4) | 22 (47.8) |
| ≥ 6 to < 12 months | 30 (23.4) | 20 (24.4) | 10 (21.7) |
| ≥ 12 months | 24 (18.8) | 10 (12.2) | 14 (30.4) |
| **Reason for T-DM1 treatment discontinuation** | | |  |
| Disease progression | 102 (79.7) | 66 (80.5) | 36 (78.3) |
| Toxicity | 21 (16.4) | 13 (15.9) | 8 (17.4) |
| Other | 5 (3.9) | 3 (3.7) | 2 (4.3) |
| **Metastatic site at start of drug therapy after T-DM1 treatment discontinuation** | | | |
| Viscera | 89 (69.5) | 62 (75.6) | 27 (58.7) |
| Skin/subcutaneous soft tissues/lymph nodes | 76 (59.4) | 46 (56.1) | 30 (65.2) |
| Bone | 53 (41.4) | 29 (35.4) | 24 (52.2) |
| CNS | 17 (13.3) | 8 (9.8) | 9 (19.6) |
| Other | 9 (7.0) | 5 (6.1) | 4 (8.7) |

Data are *n* (%) unless otherwise indicated.

^a^The study protocol states that “IHC3+ or IHC2+/ISH+ tumors are defined as HER2-positive”. However, at the case review meeting, it was determined that study patients with “IHC not performed and ISH+” who underwent anti-HER2 therapy were to be regarded as HER2-positive.

^b^Defined as Stage IV (Any T + Any N + M1) or recurrence within 6 months after the start of initial treatment.

^c^A single missing case was excluded from recurrent cases.

*CNS* central nervous system, *CR* complete response, *ECOG PS* Eastern Cooperative Oncology Group Performance Status, *HER2* human epidermal growth factor receptor 2, *IHC* immunohistochemistry, *ISH* *in situ* hybridization, *PD* progressive disease, *PR* partial response, *SD* stable disease, *T-DMI* trastuzumab emtansine.

# Supplementary Table 2 Analysis of outcomes according to subgroup

|  | **rwPFS,**  **months** | **TTF,**  **months** | **OS,**  **months** | **ORR^a^,**  **%** | **CBR,**  **%** |
| --- | --- | --- | --- | --- | --- |
| **Total population**  **(*N* = 128)** | 5.684  (4.830–6.899) | 5.552  (4.600–6.407) | 22.768  (18.168–32.427) | 23.5  (15.1–31.4) | 47.7  (38.8–56.7) |
| **Subgroup** |  |  |  |  |  |
| ECOG PS | | | | | |
| 0 (*n* = 67) | 6.669  (5.421–7.359) | 6.390  (5.224–7.162) | 28.287  (16.066–36.961) | 28.3  (17.5–41.4) | 56.7  (44.0–68.8) |
| ≥ 1 (*n* = 34) | 3.910  (2.103–5.815) | 3.844  (2.070–5.848) | 13.503  (8.345–28.747) | 21.4  (8.3–41.0) | 38.2  (22.2–56.4) |
| Visceral metastasis | | | | | |
| Yes (*n* = 89) | 5.782  (4.895–6.899) | 5.552  (4.698–6.702) | 20.731  (15.704–32.427) | 23.2  (14.6–33.8) | 48.3  (37.6–59.2) |
| No (*n* = 39) | 5.520  (3.910–7.852) | 4.862  (3.483–7.622) | 28.747  (14.357–-) | 20.7  (8.0–39.7) | 46.2  (30.1–62.8) |
| CNS metastasis | | | | | |
| Yes (*n* = 17) | 5.684  (2.070–8.214) | 5.552  (1.938–7.754) | 16.066  (8.115–-) | 28.6  (8.4–58.1) | 35.3  (14.2–61.7) |
| No (*n* = 111) | 5.782  (4.830–6.899) | 5.552  (4.632–6.702) | 25.659  (18.168–33.150) | 21.6  (13.9–31.2) | 49.5  (39.9–59.2) |
| Hormone receptor status | | | | | |
| Positive (*n* = 83) | 5.585  (4.665–6.899) | 5.552  (4.402–6.702) | 32.000  (20.074–-) | 20.3  (11.8–31.2) | 47.0  (35.9–58.3) |
| Negative (*n* = 43) | 5.815  (3.713–7.524) | 5.552  (3.483–7.097) | 14.883  (10.579–28.287) | 25.7  (12.5–43.3) | 48.8  (33.3–64.5) |
| HER2 status | | | | | |
| IHC3+ (*n* = 104) | 6.209  (5.092–7.129) | 5.815  (4.928–6.932) | 28.288  (16.164–35.910) | 25.0  (16.6–35.1) | 52.9  (42.8–62.8) |
| IHC2+ and ISH+, IHC not performed and ISH+ (*n* = 24) | 3.910  (2.070–6.669) | 3.598  (2.103–5.618) | 18.497  (13.503–-) | 10.5  (1.3–33.1) | 25.0  (9.8–46.7) |
| Number of treatment lines before T-DM1 treatment | | | | | |
| ≥ 2 (*n* = 79) | 5.947  (4.665–7.195) | 5.618  (4.238–6.702) | 24.016  (15.704–-) | 17.1  (9.2–28.0) | 46.8  (35.5–58.4) |
| < 2 (*n* = 49) | 5.388  (4.008–7.129) | 5.421  (4.041–7.097) | 22.768  (15.606–33.150) | 31.7  (18.1–48.1) | 49.0  (34.4–63.7) |
| Best response to T-DM1 treatment | | | | | |
| CR, PR (*n* = 46) | 6.669  (5.191–8.641) | 6.538  (5.027–8.674) | -  (35.910–-) | 32.4  (18.0–49.8) | 58.7  (43.2–73.0) |
| Others^b^ (*n* = 82) | 5.092  (4.008–6.867) | 4.862  (3.745–5.815) | 16.164  (14.357–24.016) | 17.6  (9.7–28.2) | 41.5  (30.7–52.9) |
| History of pertuzumab treatment | | | | | |
| Yes (*n* = 72) | 4.895  (4.008–5.782) | 4.862  (3.713–5.618) | 18.924  (14.883–28.287) | 18.0  (9.4–30.0) | 40.3  (28.9–52.5) |
| No (*n* = 56) | 7.064  (5.684–7.852) | 6.653  (5.092–7.589) | 33.150  (18.891–-) | 28.0  (16.2–42.5) | 57.1  (43.2–70.3) |
| Regimens after T-DM1 | | | | | |
| Anti-HER2 therapy (*n* = 105) | 6.341  (5.092–7.195) | 5.618  (4.632–6.932) | 28.288  (18.924–36.961) | 22.8  (14.7–32.8) | 50.5  (40.5–60.4) |
| Without anti-HER2 therapy (*n* = 23) | 4.830  (1.906–5.947) | 4.862  (1.938–5.979) | 15.277  (9.856–32.000) | 21.1  (6.1–45.6) | 34.8  (16.4–57.3) |

Data are median (95% confidence interval).

^a^*n* = 111 (population with measurable lesions)

^b^Others include: SD, non-CR/non-PD, PD, and unknown.

*CBR* clinical benefit rate, *CR* complete response, *ECOG PS* Eastern Cooperative Oncology Group Performance Status, *HER2* human epidermal growth factor receptor 2, *IHC* immunohistochemistry, *ISH* in situ hybridization, *ORR* objective response rate, *OS* overall survival, *PD* progressive disease, *PR* partial response, *rwPFS* real-world progression-free survival, *SD* stable disease, *T-DMI* trastuzumab emtansine, *TTF* time-to-treatment failure.
